# Supplementary material for: Association between life events and later depression in the population-based Heinz Nixdorf Recall study—The role of sex and optimism
Source: PLoS One. 2022 Jul 20;17(7):e0271716. doi: 10.1371/journal.pone.0271716 (PMC9299341; doi:10.1371/journal.pone.0271716)
Supplement: S1 File — (DOCX) [file pone.0271716.s001.docx]

# SUPPLEMENT

**Association between life events and later depression in the population-based Heinz Nixdorf Recall study – the role of sex and optimism**

Janine Gronewold, PhD^1^ Ela-Emsal Duman, MSc^1^ Miriam Engel, MSc^2^ Miriam Engels, MSc^3^ Johannes Siegrist, PhD^3^ Raimund Erbel, MD^2^ K-H. Jöckel, PhD^2^ Dirk M. Hermann, MD^1^

^1^Department of Neurology, University Hospital Essen, University Duisburg-Essen, Essen, Germany

^2^Institute of Medical Informatics, Biometry and Epidemiology, University of Duisburg-Essen, Germany

^3^Institute of Medical Sociology, Medical Faculty, Heinrich-Heine-University Düsseldorf, Germany


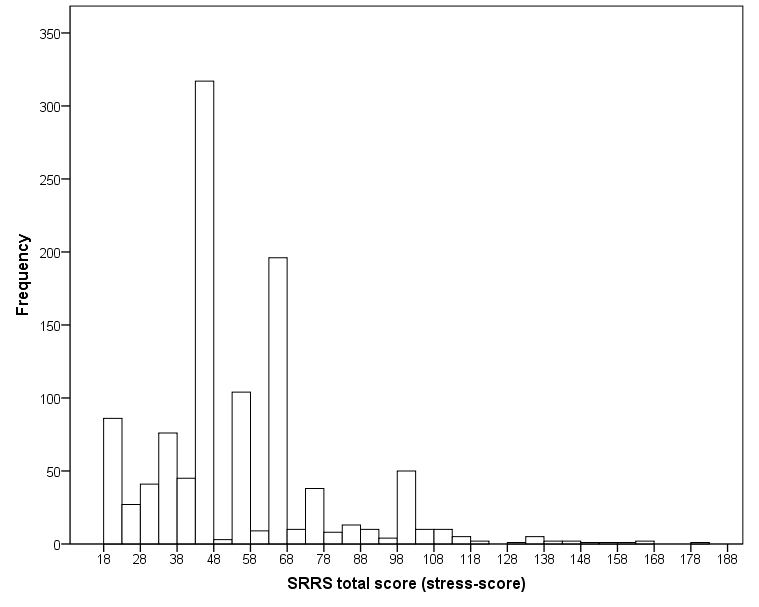


Supplemental Figure S1. Frequency distribution of the Social Readjustment Rating Scale (SRRS) total score in participants who experienced at least one important life event during the previous 6 months


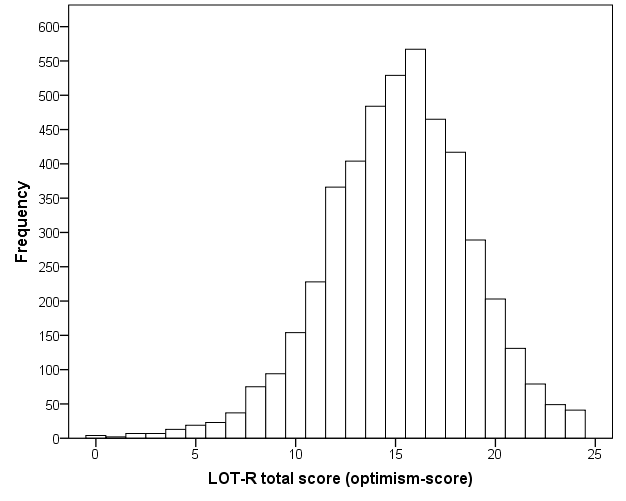


Supplemental Figure S2. Frequency distribution of the Life Orientation Test-Revised (LOT-R) total score in the total Heinz Nixdorf Recall study cohort


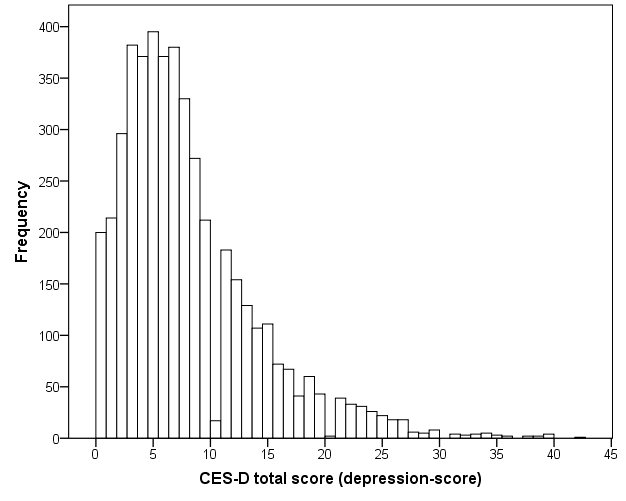
 Supplemental Figure S3. Frequency distribution of the 15 items Center for Epidemiologic Studies Depression scale (CES-D) total score in the total Heinz Nixdorf Recall study cohort

Supplemental Table S1. Presence of important life events, life event stress, and depressive symptoms stratified by age, sex, and optimism

|  | Age | | Sex | | Optimism | |  |
| --- | --- | --- | --- | --- | --- | --- | --- |
|  | >65 years | ≤65 years | Men | Women | Optimists | Pessimists |  |
| Presence of important life event,  n (%) | 220 (17.6) | 900 (25.3) | 491 (20.5) | 629 (26.0) | 175 (22.1) | 932 (23.9) | |
| Life event stress, score, median (Q1;Q3) | 44.0 (44.0;63.0) | 45.0 (39.0;63.0) | 45.0 (39.0;63.0) | 44.0 (44.0;63.0) | 53.0 (44.0;63.0) | 45.0 (39.0;63.0) | |
| Depression score, median (Q1;Q3) | 7.0 (4.0;11.0) | 7.0 (4.0;11.0) | 6.0 (3.0;9.0) | 7.0 (4.0;12.0) | 4.0 (2.0;7.0) | 7.0 (4.0;12.0) | |

Supplemental Table S2. Frequency of important life events according to the Social Readjustment Rating Scale (SRRS) in its original order stratified by age

| Life event | | Life Change Unit | >65 years | ≤65 years |
| --- | --- | --- | --- | --- |
|  |  |  | Number (%) | Number (%) |
| 1 | Death of spouse | 100 | 16 (1.3) | 22 (0.6) |
| 2 | Divorce | 73 | 2 (0.2) | 20 (0.6) |
| 3 | Marital separation | 65 | 1 (0.1) | 32 (0.9) |
| 4 | Jail term | 63 | 0 (0.0) | 1 (0.1) |
| 5 | Death of close family member | 63 | 49 (3.9) | 161 (4.5) |
| 6 | Personal injury or illness | 53 | 26 (2.1) | 98 (2.8) |
| 7 | Marriage | 50 | 0 (0.0) | 2 (0.1) |
| 8 | Fired at work | 47 | 1 (0.1) | 35 (1.0) |
| 9 | Marital reconciliation | 45 | 1 (0.1) | 0 (0.0) |
| 10 | Retirement | 45 | 3 (0.2) | 53 (1.5) |
| 11 | Change in health of family member | 44 | 73 (5.8) | 237 (6.7) |
| 12 | Pregnancy | 39 | 0 (0.0) | 0 (0.0) |
| 13 | Sex difficulties | 39 | 0 (0.0) | 0 (0.0) |
| 14 | Gain of new family member | 39 | 6 (0.5) | 24 (0.7) |
| 15 | Business readjustment | 39 | 0 (0.0) | 0 (0.0) |
| 16 | Change in financial state | 38 | 4 (0.3) | 27 (0.8) |
| 17 | Death of close friend | 37 | 12 (1.0) | 47 (1.3) |
| 18 | Change to different line of work | 36 | 0 (0.0) | 25 (0.7) |
| 19 | Change in number of arguments with spouse | 35 | 1 (0.1) | 25 (0.7) |
| 20 | Mortgage over $10,000 | 31 | 0 (0.0) | 1 (0.1) |
| 21 | Foreclosure of mortgage or loan | 30 | 0 (0.0) | 0 (0.0) |
| 22 | Change in responsibilities at work | 29 | 0 (0.0) | 9 (0.3) |
| 23 | Son or daughter leaving home | 29 | 6 (0.5) | 16 (0.4) |
| 24 | Trouble with in-laws | 29 | 7 (0.6) | 18 (0.5) |
| 25 | Outstanding personal achievement | 28 | 0 (0.0) | 1 (0.1) |
| 26 | Wife begin or stop work | 26 | 0 (0.0) | 3 (0.1) |
| 27 | Begin or end school | 26 | 0 (0.0) | 0 (0.0) |
| 28 | Change in living conditions | 25 | 5 (0.4) | 37 (1.0) |
| 29 | Revision of personal habits | 24 | 0 (0.0) | 2 (0.1) |
| 30 | Trouble with boss | 23 | 0 (0.0) | 5 (0.1) |
| 31 | Change in work hours or conditions | 20 | 2 (0.2) | 71 (2.0) |
| 32 | Change in residence | 20 | 8 (0.6) | 61 (1.7) |
| 33 | Change in schools | 20 | 0 (0.0) | 0 (0.0) |
| 34 | Change in recreation | 19 | 0 (0.0) | 0 (0.0) |
| 35 | Change in church activities | 19 | 0 (0.0) | 0 (0.0) |
| 36 | Change in social activities | 18 | 0 (0.0) | 0 (0.0) |
| 37 | Mortgage or loan less than $50,000 | 17 | 0 (0.0) | 0 (0.0) |
| 38 | Change in sleeping habits | 16 | 0 (0.0) | 0 (0.0) |
| 39 | Change in number of family get-togethers | 15 | 0 (0.0) | 1 (0.1) |
| 40 | Change in eating habits | 15 | 0 (0.0) | 0 (0.0) |
| 41 | Vacation | 13 | 0 (0.0) | 0 (0.0) |
| 42 | Christmas | 12 | 0 (0.0) | 0 (0.0) |
| 43 | Minor violation of laws | 11 | 0 (0.0) | 0 (0.0) |

Supplemental Table S3. Frequency of important life events according to the Social Readjustment Rating Scale (SRRS) in its original order stratified by sex

| Life event | | Life Change Unit | Men | Women |
| --- | --- | --- | --- | --- |
|  |  |  | Number (%) | Number (%) |
| 1 | Death of spouse | 100 | 8 (0.3) | 30 (1.2) |
| 2 | Divorce | 73 | 8 (0.3) | 14 (0.6) |
| 3 | Marital separation | 65 | 15 (0.6) | 18 (0.7) |
| 4 | Jail term | 63 | 0 (0.0) | 1 (0.1) |
| 5 | Death of close family member | 63 | 101 (3.2) | 109 (4.5) |
| 6 | Personal injury or illness | 53 | 68 (2.8) | 56 (2.3) |
| 7 | Marriage | 50 | 0 (0.0) | 2 (0.1) |
| 8 | Fired at work | 47 | 15 (0.6) | 21 (0.9) |
| 9 | Marital reconciliation | 45 | 1 (0.1) | 0 (0.0) |
| 10 | Retirement | 45 | 35 (1.5) | 21 (0.9) |
| 11 | Change in health of family member | 44 | 103 (4.3) | 207 (8.6) |
| 12 | Pregnancy | 39 | 0 (0.0) | 0 (0.0) |
| 13 | Sex difficulties | 39 | 0 (0.0) | 0 (0.0) |
| 14 | Gain of new family member | 39 | 10 (0.4) | 20 (0.8) |
| 15 | Business readjustment | 39 | 0 (0.0) | 0 (0.0) |
| 16 | Change in financial state | 38 | 12 (0.5) | 19 (0.8) |
| 17 | Death of close friend | 37 | 25 (1.0) | 34 (1.4) |
| 18 | Change to different line of work | 36 | 18 (0.8) | 7 (0.3) |
| 19 | Change in number of arguments with spouse | 35 | 9 (0.4) | 17 (0.7) |
| 20 | Mortgage over $10,000 | 31 | 1 (0.1) | 0 (0.0) |
| 21 | Foreclosure of mortgage or loan | 30 | 0 (0.0) | 0 (0.0) |
| 22 | Change in responsibilities at work | 29 | 9 (0.4) | 0 (0.0) |
| 23 | Son or daughter leaving home | 29 | 8 (0.3) | 14 (0.6) |
| 24 | Trouble with in-laws | 29 | 8 (0.3) | 17 (0.7) |
| 25 | Outstanding personal achievement | 28 | 0 (0.0) | 1 (0.1) |
| 26 | Wife begin or stop work | 26 | 3 (0.1) | 0 (0.0) |
| 27 | Begin or end school | 26 | 0 (0.0) | 0 (0.0) |
| 28 | Change in living conditions | 25 | 14 (0.6) | 28 (1.2) |
| 29 | Revision of personal habits | 24 | 1 (0.1) | 1 (0.1) |
| 30 | Trouble with boss | 23 | 3 (0.1) | 2 (0.1) |
| 31 | Change in work hours or conditions | 20 | 34 (1.4) | 39 (1.6) |
| 32 | Change in residence | 20 | 24 (1.0) | 45 (1.9) |
| 33 | Change in schools | 20 | 0 (0.0) | 0 (0.0) |
| 34 | Change in recreation | 19 | 0 (0.0) | 0 (0.0) |
| 35 | Change in church activities | 19 | 0 (0.0) | 0 (0.0) |
| 36 | Change in social activities | 18 | 0 (0.0) | 0 (0.0) |
| 37 | Mortgage or loan less than $50,000 | 17 | 0 (0.0) | 0 (0.0) |
| 38 | Change in sleeping habits | 16 | 0 (0.0) | 0 (0.0) |
| 39 | Change in number of family get-togethers | 15 | 0 (0.0) | 1 (0.1) |
| 40 | Change in eating habits | 15 | 0 (0.0) | 0 (0.0) |
| 41 | Vacation | 13 | 0 (0.0) | 0 (0.0) |
| 42 | Christmas | 12 | 0 (0.0) | 0 (0.0) |
| 43 | Minor violation of laws | 11 | 0 (0.0) | 0 (0.0) |

Supplemental Table S4. Frequency of important life events according to the Social Readjustment Rating Scale (SRRS) in its original order stratified by optimism

| Life event | | Life Change Unit | Optimists | Pessimist |
| --- | --- | --- | --- | --- |
|  |  |  | Number (%) | Number (%) |
| 1 | Death of spouse | 100 | 6 (0.8) | 32 (0.8) |
| 2 | Divorce | 73 | 2 (0.3) | 20 (0.5) |
| 3 | Marital separation | 65 | 3 (0.4) | 30 (0.8) |
| 4 | Jail term | 63 | 1 (0.1) | 0 (0.0) |
| 5 | Death of close family member | 63 | 38 (4.8) | 169 (4.3) |
| 6 | Personal injury or illness | 53 | 26 (3.3) | 95 (2.4) |
| 7 | Marriage | 50 | 1 (0.1) | 1 (0.1) |
| 8 | Fired at work | 47 | 4 (0.5) | 32 (0.8) |
| 9 | Marital reconciliation | 45 | 0 (0.0) | 1 (0.1) |
| 10 | Retirement | 45 | 10 (1.3) | 46 (1.2) |
| 11 | Change in health of family member | 44 | 45 (5.7) | 262 (6.7) |
| 12 | Pregnancy | 39 | 0 (0.0) | 0 (0.0) |
| 13 | Sex difficulties | 39 | 0 (0.0) | 0 (0.0) |
| 14 | Gain of new family member | 39 | 5 (0.6) | 25 (0.6) |
| 15 | Business readjustment | 39 | 0 (0.0) | 0 (0.0) |
| 16 | Change in financial state | 38 | 6 (0.8) | 25 (0.6) |
| 17 | Death of close friend | 37 | 6 (0.8) | 52 (1.3) |
| 18 | Change to different line of work | 36 | 2 (0.3) | 23 (0.6) |
| 19 | Change in number of arguments with spouse | 35 | 4 (0.5) | 22 (0.6) |
| 20 | Mortgage over $10,000 | 31 | 0 (0.0) | 1 (0.1) |
| 21 | Foreclosure of mortgage or loan | 30 | 0 (0.0) | 0 (0.0) |
| 22 | Change in responsibilities at work | 29 | 1 (0.1) | 8 (0.2) |
| 23 | Son or daughter leaving home | 29 | 1 (0.1) | 21 (0.5) |
| 24 | Trouble with in-laws | 29 | 4 (0.5) | 21 (0.5) |
| 25 | Outstanding personal achievement | 28 | 1 (0.1) | 0 (0.0) |
| 26 | Wife begin or stop work | 26 | 0 (0.0) | 3 (0.1) |
| 27 | Begin or end school | 26 | 0 (0.0) | 0 (0.0) |
| 28 | Change in living conditions | 25 | 8 (1.0) | 34 (0.9) |
| 29 | Revision of personal habits | 24 | 0 (0.0) | 2 (0.1) |
| 30 | Trouble with boss | 23 | 1 (0.1) | 4 (0.1) |
| 31 | Change in work hours or conditions | 20 | 12 (1.5) | 60 (1.5) |
| 32 | Change in residence | 20 | 10 (1.3) | 58 (1.5) |
| 33 | Change in schools | 20 | 0 (0.0) | 0 (0.0) |
| 34 | Change in recreation | 19 | 0 (0.0) | 0 (0.0) |
| 35 | Change in church activities | 19 | 0 (0.0) | 0 (0.0) |
| 36 | Change in social activities | 18 | 0 (0.0) | 0 (0.0) |
| 37 | Mortgage or loan less than $50,000 | 17 | 0 (0.0) | 0 (0.0) |
| 38 | Change in sleeping habits | 16 | 0 (0.0) | 0 (0.0) |
| 39 | Change in number of family get-togethers | 15 | 0 (0.0) | 1 (0.1) |
| 40 | Change in eating habits | 15 | 0 (0.0) | 0 (0.0) |
| 41 | Vacation | 13 | 0 (0.0) | 0 (0.0) |
| 42 | Christmas | 12 | 0 (0.0) | 0 (0.0) |
| 43 | Minor violation of laws | 11 | 0 (0.0) | 0 (0.0) |
