# Supplementary material for: Association between life events and later depression in the population-based Heinz Nixdorf Recall study—The role of sex and optimism
Source: PLoS One. 2022 Jul 20;17(7):e0271716. doi: 10.1371/journal.pone.0271716 (PMC9299341; doi:10.1371/journal.pone.0271716)
Supplement: Attachment — Submitted filename: ResponseToReviewers_Revision2.docx [file pone.0271716.s003.docx]

Gronewold J, et al.

**‘Association between life events and later depression in the population-based Heinz Nixdorf Recall study – the role of sex and optimism’ (MS ID: PONE-D-21-02934R1)**

**Responses to reviewers**

**Please note that all new/revised text is highlighted in yellow in the manuscript.**

# Reviewer #1

**The authors have addressed the previous points that were raised, and the re-working of the introduction and discussion makes the message much clearer. The inclusion of the additional information in the supplementary tables is also very useful. I have one main question arising from the inclusion of these new tables:**

**Table S4 shows the frequency of different life events stratified by optimism, and shows that the cut off for determining whether a participant is an optimist or a pessimist results in many more pessimists than optimists (i.e., 0.8% of the optimist group is 6 participants, but 0.8% of the pessimist group is 32 people). Is it possible that the stronger association between life event stress and depression in the pessimist group is due to increased statistical power because the sample size is larger? To put it another way, does the association look smaller in the optimist group because of lower power? If this is a possibility, how would it affect the conclusions?**

🡪 In Table S4, the number and percentage of optimists and pessimists reporting different life events is shown. 6 participants of the optimist group (0.8%) reported that they experienced the life event “death of a spouse”, which was reported by the same percentage of participants from the pessimist group (0.8%, n=32). Since we defined optimists as subjects with Life Orientation Test-Revised score above the upper quartile (>Q3) and pessimists as subjects with Life Orientation Test-Revised score below the upper quartile (≤Q3), we have a smaller sample size in the optimist group (n=781) than in the pessimist group (n=3820). Even though we thus have higher statistical power in the pessimist group, resulting in narrower confidence intervals and lower p-values, the effect estimates for the association of life event stress and depression clearly differ between optimists and pessimist with the unstandardized regression weight from linear regression being 0.9 in optimists and 2.3 in pessimists (Table 3). According to up-to-date statistical recommendations, relevant effect modification is assumed in case of a difference in effect estimates of more than 10%. In our study, we have a difference of 55%, which clearly indicates that the effect of life events on depression is modified by the character trait optimism vs pessimism. This is also supported by the observed statistically highly significant interaction term (p<0.001) between life event stress and the character trait optimism vs pessimism (line 325). Consequently, the different sample size of optimists and pessimists does not affect our conclusion of a stronger association between life event stress and depression pessimists.

**Aside from that question, I have just a few minor comments after my second reading of the manuscript. In the following comments, line numbers refer to the line numbers in the revised manuscript.**

**1. line 70: "self-report screenings which are often not feasible in large population based studies" - I think the authors are arguing that self-report measures are usually used in larger studies because structured interviews are usually not feasible, but the wording makes it sound like it's the self-report measures that are not feasible.**

🡪 The reviewer is completely right, that sentence was misleading, we changed it to “The minority of population-based studies assessed depression with structured diagnostic interviews because – compared with self-report screenings – structured diagnostic interviews require a lot more time and psychiatrically trained staff and thus are often not feasible in large population-based studies designed to address multiple research questions.

**2. lines 151-152: I think it would be good to include the details of the second-coding and reliability analysis for the life event scoring in here, so that readers can see that the scoring of life events was carried out more rigorously.**

🡪 We now included details of the second-coding and reliability analysis as suggested (line 152/153, line 172).

**3. line 175: "optimism is regarded AS a stable trait" - the word as is missing**

🡪 Changed as suggested.

**4. line 327: stronger influence of stress on optimists compared to pessimists - this appears to be the opposite of what is claimed elsewhere**

🡪 That was a mistake, we changed it to “This interaction revealed the stronger influence of life event stress on pessimists compared to optimists.”

**5. line 392-393: an argument is made about some life events having more impact on men vs. women, and vice versa. A reference is needed here**

🡪 Reference inserted as suggested.
